# Hyperpolarization-Activated Cyclic Nucleotide-Gated Channels: An Emerging Role in Neurodegenerative Diseases

**DOI:** 10.3389/fnmol.2019.00141

**Published:** 2019-06-05

**Authors:** Xiaoli Chang, Jun Wang, Hong Jiang, Limin Shi, Junxia Xie

**Affiliations:** ^1^Department of Physiology, Shandong Provincial Key Laboratory of Pathogenesis and Prevention of Neurological Disorders and State Key Disciplines: Physiology, Medical College of Qingdao University, Qingdao, China; ^2^Institute of Brain Science and Disease, Qingdao University, Qingdao, China

**Keywords:** Parkinson’s disease, Alzheimer’s disease, HCN channels, *I*_h_, amyotrophic lateral sclerosis, spinal muscular atrophy

## Abstract

Neurodegenerative diseases such as Parkinson’s disease (PD), Alzheimer’s disease (AD), amyotrophic lateral sclerosis (ALS), and spinal muscular atrophy (SMA) are chronic, progressive, and age-associated neurological disorders characterized by neuronal deterioration in specific brain regions. Although the specific pathological mechanisms underlying these disorders have remained elusive, ion channel dysfunction has become increasingly accepted as a potential mechanism for neurodegenerative diseases. Hyperpolarization-activated cyclic nucleotide-gated (HCN) channels are encoded by the *HCN1-4* gene family and conduct the hyperpolarization-activated current (*I*_h_). These channels play important roles in modulating cellular excitability, rhythmic activity, dendritic integration, and synaptic transmission. In the present review, we first provide a comprehensive picture of the role of HCN channels in PD by summarizing their role in the regulation of neuronal activity in PD-related brain regions. Dysfunction of *I*_h_ may participate in 1-methyl-4-phenylpyridinium (MPP^+^)-induced toxicity and represent a pathogenic mechanism in PD. Given current reports of the critical role of HCN channels in neuroinflammation and depression, we also discussed the putative contribution of HCN channels in inflammatory processes and non-motor symptoms in PD. In the second section, we summarize how HCN channels regulate the formation of β-amyloid peptide in AD and the role of these channels in learning and memory. Finally, we briefly discuss the effects of HCN channels in ALS and SMA based on existing discoveries.

## Introduction

Hyperpolarization-activated cyclic nucleotide-gated (HCN) channels are voltage-gated channels encoded by the *HCN1-4* gene family. These channels are primarily expressed in the heart and in the central and peripheral nervous systems (Moosmang et al., 1999, 2010; Notomi and Shigemoto, 2010). HCN channels conduct K^+^ and Na^+^ ions at a ratio of 3:1 to 5:1. They are activated by hyperpolarization of membrane voltage to -50 mV or below, and conduct the hyperpolarization-activated current, termed *I*_f_ in heart and *I*_h_ in neurons (Brown et al., 1979; Difrancesco, 1993; Ludwig et al., 1998; Santoro et al., 1998). cAMP can regulate the voltage-dependent activation of HCN channels in a subtype-specific manner, with HCN2 and HCN4 channels being highly susceptible to this molecule (He et al., 2014). Downregulation of cAMP suppresses *I*_h_ and shifts the HCN activation curve to lower voltage values (Biel et al., 2009). HCN channels play essential roles in the modulation of neuronal excitability, rhythmic neuronal activity, dendritic integration, and synaptic transmission, thus mediate multiple physiological functions. For example, HCN channels are able to regulate sleep and wakefulness (Kanyshkova et al., 2009; Zobeiri et al., 2018), learning and memory (Nolan et al., 2003; Nolan et al., 2004), and somatic sensation (Emery and Mcnaughton, 2011; Ding et al., 2018). Indeed, dysfunction of HCN channels is closely associated with several pathophysiological states, including epilepsy (Dibbens et al., 2010; Nava et al., 2014), neuropathic pain (Emery et al., 2012; Tibbs et al., 2016), and inflammatory pain (Emery et al., 2012; Stadler et al., 2014). Recent studies have also shown that HCN channels play a significant but complex role in neurodegenerative diseases.

Neurodegenerative diseases are chronic, progressive, and age-associated disorders, characterized by selective loss of neurons in specific brain regions. As the global population increases, the incidence of these diseases increases accordingly, and thus neurodegenerative diseases have become a serious public health issue. Nevertheless, their etiology and pathogenesis have not yet been fully elucidated, which hinders the identification of key therapeutics, and the evaluation of their potential application as therapeutic. The pathogenesis of neurological disease is closely associated with the dysfunction of neuronal excitability, rhythmicity, and signaling, which are generated and modulated by specific sets of proteins, including ion channels. HCN channels are widely expressed in the basal ganglia and hippocampus, where they control the electrical activities of substantia nigra pars compacta (SNc) dopaminergic neurons and hippocampal glutamatergic neurons (Okamoto et al., 2006; Sinha and Narayanan, 2015). In agreement with these findings, HCN channel dysfunction has been implicated in PD and AD (Chan et al., 2011; Saito et al., 2012). Changes of HCN channels are also observed in ALS and SMA (Lai et al., 2018; Silbernagel et al., 2018). Therefore, in this review, we focus on the roles of HCN channels in the pathogenesis of PD, AD, and other neurodegenerative diseases. We also discuss whether modulation of these channels could provide a new therapeutic target for the alleviation of symptoms related to these neurodegenerative diseases.

## HCN Channels and PD

Parkinson’s disease is the second most common neurodegenerative disorder, characterized by a progressive degeneration of dopaminergic neurons in the SNc. PD patients often suffer from an array of motor impairments, including rigidity, resting tremor, postural instability, and bradykinesia. Non-motor symptoms such as olfactory loss, anxiety and depression, sleep abnormalities, and constipation are also present during early stages of the disease (Singaram et al., 1995; Weisskopf et al., 2003; Bohnen et al., 2007; Postuma et al., 2017; Del Rey et al., 2018). Dopaminergic neurons in SNc send neurological projections to several brain regions and help to chemically differentiate the direct and indirect pathways. Previous reports have shown that HCN channels are widely expressed in multiple basal ganglia such as the SNc, the globus pallidus (GP), and the STN. Moreover, HCN channels are involved in the regulation of electrical activities of these neurons under physiological and pathological conditions (Santoro et al., 2000; Moosmang et al., 2010). To date, studies on HCN channels and PD have mainly focused on the following aspects: (1) alteration of HCN channel expression and function in PD animal models, (2) potential involvement of HCN channels in the neurotoxic effects of MPP^+^, and (3) HCN channels as a mechanism for the selective vulnerability of SNc neurons. Given current reports of the critical role of HCN channels in neuroinflammation and depression, we also discuss their possible involvement in the inflammatory processes and non-motor symptoms of PD.

## HCN Channels Regulate the Electrical Activities of Neurons in the Basal Ganglia

In the 1980s, *I*_h_ was first recorded in SNc dopaminergic neurons (Lacey et al., 1987, 1989). The current was observed to become slowly activated at approximately -70 mV and was fully activated at -129 to -140 mV (Lacey et al., 1989; Mercuri et al., 1995). The current was specifically blocked by ZD7288 within a specific concentration range (<100 μM) (Harris and Constanti, 1995; Seutin et al., 2001; Neuhoff et al., 2002). Later, mRNA expression of HCN2–HCN4 was examined in SNc dopaminergic neurons using single-cell RT-mPCR and *in situ* hybridization (Franz et al., 2000; Santoro et al., 2000), showing that both HCN2 and HCN4 are expressed at relatively higher levels than HCN3 (Mercuri et al., 1995; Franz et al., 2000; Gambardella et al., 2012). Under current-clamp conditions, dopaminergic neurons exhibit a pronounced rebound depolarization (sag) mediated by *I*_h_ in response to a series of hyperpolarizing current pulses. The presence of *I*_h_ or sag has been used as a key criterion for the identification of dopaminergic neurons (Grace and Onn, 1989). In these neurons, *I*_h_ has a high density of expression around the axon-bearing dendrites, and is reduced with distance from the axon origin, which may affect synaptic integration (Engel and Seutin, 2015). Neuhoff et al. (2002) further reported that there were significant differences in HCN channel density in various subpopulations of dopaminergic neurons. Calbindin-negative SNc dopaminergic neurons, which were more vulnerable to neurotoxins, possessed a larger sag amplitude and *I*_h_ density than calbindin-positive neurons (Neuhoff et al., 2002). Neurons in the GP also respond to series of hyperpolarizing current pulses with a drop in current (sag). Studies have shown that HCN1–4 channels are expressed in the GP and STN. Among them, HCN2 is the major isoform in the GP (Chan et al., 2004), while HCN2 and HCN3 are more highly expressed in the STN (Atherton et al., 2010).

HCN channels have been reported to regulate the electrical activities of neurons in basal ganglia. SNc dopaminergic neurons display two predominant types of firing patterns *in vivo*: tonic irregular single-spike activity and phasic burst activity. *In vitro*, they only exhibit slow and regular spontaneous firing activity. The spontaneous firing activity of dopaminergic neurons was significantly inhibited after application of the HCN channels blocker ZD7288 (<100 μM) in acutely prepared mouse or rat midbrain slices (Seutin et al., 2001; Neuhoff et al., 2002; Okamoto et al., 2006). Chan et al. (2007) further observed that ZD7288 (50 μM) inhibited spontaneous firing activity in juvenile wild-type SNc dopaminergic neurons (less than postnatal 21 days), and was able to completely block firing in adult Cav1.3^-/-^ SNc dopaminergic neurons. Pharmacological blockade of HCN channels also promoted burst firing in SNc dopaminergic neurons of mice, which led to the release of a large amount of dopamine (Mrejeru et al., 2011).

HCN channels also make an important contribution to the autonomous pacemaking in GP neurons (Chan et al., 2004; Surmeier et al., 2005). Application of ZD7288 (50 μM) or Cs^+^ in acutely prepared mouse brain slices led to hyperpolarization and reduced firing rate and regularity (Chan et al., 2004). Subsequently, a novel computational model of GPe neurons confirmed that when HCN channels were blocked, the pacemaker activity of GPe neurons was reduced or abolished (Merrisonhort and Borisyuk, 2013). However, HCN channels could regulate firing of GP neurons bidirectionally *in vivo* (Chen et al., 2015). Using single-unit extracellular recordings, Chen et al. observed that micro-pressure ejection of ZD7288 and Cs^+^ decreased the frequency of spontaneous firing in 21 out of 40 GP neurons recorded, but increased the firing rate in another 18 neurons. Similar results were obtained when using 8-Br-cAMP, an activator of HCN channels (Chen et al., 2015). Since GP neurons consist of two types of neurons, GP-TI and GP-TA (Mallet et al., 2012), the bidirectional modulation of HCN channel firing activity may be a result of the interplay between these two types of neurons. As for the effect of HCN channels on pacemaking activity in the STN neurons, previous articles have reported that the effect was negligible in rat brain slices (Bevan and Wilson, 1999; Do and Bean, 2003). However, Deng et al. (2015) revealed that HCN channels could bidirectionally regulate the firing of STN neurons *in vivo* by using single-unit extracellular recordings, which was similar to their previously published report in the GP.

HCN channels also regulate the oscillatory activity of basal ganglia neurons. Oscillation activities, mainly including alpha (8–12 Hz), beta (11–30 Hz), delta (1–3 Hz), and theta (2–7 Hz)-frequency bands, are prominent features of the neuronal network and are closely related to many brain behavioral and functional states (Hutchison et al., 2004). Theta activity has been reported to lead to a paroxysmal increase in freezing behavior in PD patients (Follett and Torresrussotto, 2012; Hu et al., 2018). HCN channels reportedly contribute to theta frequency membrane resonance in hyperpolarized mammalian SNc neurons, which may be involved in theta oscillation (Xue et al., 2012). This membrane resonance in the hyperpolarized potential ranging from -60 to -80 mV was completely abolished by application of ZD7288 (10 μM) (Xue et al., 2012). In the late stage PD, excessive synchronized oscillation and high-frequency bursts often occur in STN neurons (Obeso et al., 2000; Bevan et al., 2002; Syed et al., 2012). A recent study showed that the resonance in rat STN was also mediated by HCN channels, and application of ZD7288 (20 μM) could abolish it (Yan et al., 2012).

## Alteration of HCN Channel Expression and Function in PD Animal Models

Functional changes in HCN channels within the basal ganglia have been observed in several animal models of PD. Previous studies have shown a progressive downregulation of HCN channels in SNc neurons in MitoPark mice (Good et al., 2011), a transgenic model in which a mitochondrial mutation caused parkinsonism (Ekstrand et al., 2007). MitoPark mice exhibited reduced current amplitude and a more negative *I*_h_ activation curve at postnatal 7–8 weeks, whereas motor dysfunction was not observed until 12 weeks. Therefore, HCN channel downregulation in SNc dopaminergic neurons may be one of the earliest physiological changes related to PD in MitoPark mice (Good et al., 2011). The finding that functional changes occur in SNc dopaminergic neurons prior to the onset of distinct behavioral symptoms in animal models of PD and Parkinsonian patients suggests that *I*_h_ could play an important role in the early stages of disease. Consistent with the above results, reduced *I*_h_ amplitude and density were recently observed in SNc dopaminergic neurons in spontaneous α-synuclein overexpressing rats (Guatteo et al., 2016).

The amplitude of *I*_h_ in GP neurons was also significantly reduced in acute reserpine-treated mice (Chan et al., 2011). Similar results were obtained in mice and rats as a result of chronic injection of 6-OHDA, a classic neurotoxin that causes PD (Chan et al., 2011). In all of these models, the downregulation of HCN channel expression/activity contributed to the progressive decline in the autonomous pacemaker activity of GPe neurons. They further demonstrated that the protein and mRNA levels of the four HCN subunits were reduced in the GPe, with HCN2 being the most affected. Moreover, dopamine depletion induced a significant reduction in tetratricopeptide repeat-containing TRIP8b mRNA. TRIP8b, a brain-specific cytoplasmic protein and an HCN channel auxiliary subunit in the mammalian brain, influences HCN channel surface expression and regulates their gating and kinetics (Zolles et al., 2009; Bankston et al., 2012). Thus, these results suggested that both transcriptional and trafficking-related mechanisms may be involved in the downregulation of HCN channels, producing a progressive loss of autonomous pacemaking in GPe neurons and exacerbating their rhythmic bursting (Chan et al., 2011).

Meurers and Dziewczapolski (2009) also studied the role of HCN channels in the GPi. They demonstrated that HCN3 mRNA was selectively upregulated by twofold in the entopeduncular nucleus/GPi of rats and mice with 6-OHDA-induced parkinsonism. In agreement with this change in mRNA expression, both HCN3 current amplitude and neuronal excitability were significantly increased in the lesioned animals (Meurers and Dziewczapolski, 2009). These data indicated a potential association of HCN channels with altered excitability of basal ganglia output neurons in PD. In addition, the role of HCN channels in the GP may depend on the subtype and distribution environment of the channels.

## Potential Involvement of HCN Channels in the Neurotoxic Effects of MPP^+^

MPP^+^ is a traditional neurotoxin that can induce Parkinsonian syndrome in experimental primates and rodents. It is converted from the pro-toxin MPTP to MPP^+^ and then selectively uptaken by SNc dopaminergic neurons through the DAT. Once inside the cell, MPP^+^ primarily inhibits mitochondrial complex I, leading to ATP depletion, oxidative stress, and eventual cell death (Nicklas et al., 1985; Smeyne and Jackson-Lewis, 2005; Huang et al., 2017). In addition to the classical mitochondrial impairment mechanism, several studies have also described the effects of MPP^+^ on neuronal electrophysiological activities. Acute application of MPP^+^ on midbrain slices led to hyperpolarization of SNc dopaminergic neurons and reduced their firing rate (Liss et al., 2005; Masi et al., 2013; Yee et al., 2014). However, the ionic mechanisms of this toxin on neuronal electrophysiological properties remain controversial. Previous work has attributed the effects of MPP^+^ to the opening of ATP-sensitive potassium (K-ATP) channels, which is supported by the observation that MPP^+^ caused mitochondrial failure and ATP depletion (Liss et al., 2005). Subsequently, Masi et al. (2013) demonstrated that MPP^+^ still led to a rapid hyperpolarization along with a reduction in spontaneous firing activity, even after preincubation with the K-ATP channel blocker glybenclamide. They further proposed that the effects of MPP^+^ were mediated by HCN channels (Masi et al., 2013), specifically that MPP^+^ inhibited *I*_h_, shifted the *I*_h_ activation curve toward negative potentials, and slowed the *I*_h_ activation kinetics. These effects occurred under K-ATP channel blockade and in the presence of 2 mM ATP, indicating that it was independent of mitochondrial mechanisms. Thus, they speculated that MPP^+^ may directly interact with HCN channels (Masi et al., 2013). However, Yee et al. (2014) reported that MPP^+^ inhibited the activity of dopaminergic neurons in distinct stages through different mechanisms. The early phase of inhibition was dependent on D2 autoreceptors, whereas the late phase was due to the activation of K-ATP channels. Although *I*_h_ was reduced by MPP^+^ in their study, pharmacological blockade of HCN channels did not prevent the inhibitory effects of MPP^+^. Thus, the inhibition of *I*_h_ by MPP^+^ may promote hyperpolarization through activation of some channels, such as K-ATP channels (Yee et al., 2014).

In summary, current literature indicates that MPP^+^ has acute inhibitory actions on nigral dopaminergic neurons. Although independent groups have explored ionic mechanisms in this context, results remain controversial. One should note that these studies used inconsistent concentrations of MPP^+^ (range from 10 to 50 μM), electrophysiological recording methods, and drug administration methods. Further studies are required to assess the impact of HCN channels on the neurotoxicity of MPP^+^.

## HCN Channels May Be an Ion Channel Mechanism Underlying the Selective Vulnerability of SNc Neurons

A hallmark of PD is the selective vulnerability of SNc dopaminergic neurons to damage and cell death and is contrasted with the relative resistance of dopaminergic neurons in the neighboring VTA to the disease (Ekstrand et al., 2007; Blesa and Przedborski, 2014; Brichta and Greengard, 2014). However, the mechanisms underlying this selective vulnerability remain unclear. The genetic profile of these regions only differs by 1–3% (Greene et al., 2005), indicating that the genetic factors may not account for this discrepancy. Recent research has begun to explore the role of electrophysiological determinants, proposing that specific types of ion channels, including HCN channels, may be the basis for this selective vulnerability (Table 1) (Dragicevic et al., 2015).

**Table 1:** Comparison of electrophysiological parameters of dopaminergic neurons in the SNc and VTA.

Electrophysiological parameters	SNc ( 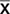 ± SEM)	VTA ( 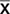 ± SEM)	Selected references
Body size (μm^2^)	176.29 ± 5.84	113.18 ± 3.57	Krashia et al., 2017
Rm (MΩ)	227 ± 10	697 ± 99	Krashia et al., 2017
Cm (pF)	57 ± 2	44 ± 4	Krashia et al., 2017
Sag (mV)	37.3 ± 0.72	31.0 ± 1.72	Neuhoff et al., 2002
*I*_h_ (pA)	299 ± 14	45 ± 9	Krashia et al., 2017
Firing frequency (Hz)	2.1 ± 0.2	1.5 ± 0.1	Werkman et al., 2001
	Significantly inhibited after pharmacologically blocking *I*_h_	Negligible	Neuhoff et al., 2002; Krashia et al., 2017
EPSP	Obviously enhanced after pharmacologically blocking *I*_h_	Negligible	Masi et al., 2013
IPSP	Significantly inhibited after pharmacologically blocking *I*_h_	Negligible	Masi et al., 2013
SCR	Remarkably activated after pharmacologically blocking *I*_h_	Negligible	Carbone et al., 2017


*Rm, membrane resistance; Cm, capacitance values; EPSP, excitatory post-synaptic potential; IPSP, inhibitory post-synaptic potential; SCR, somatic calcium response.*

Compared to VTA dopaminergic neurons, SNc neurons exhibit a larger cell body size, lower Rm, higher Cm (Krashia et al., 2017), faster firing frequency (Werkman et al., 2001; Krashia et al., 2017), and a distinct sag in response to a series of hyperpolarizing current pulses (Neuhoff et al., 2002), which appeared to correlate with the larger *I*_h_ magnitude. Indeed, the distribution of HCN channels in midbrain dopaminergic neurons is consistent with a ventro-dorsal gradient. In other words, SNc neurons show a larger average *I*_h_ than VTA neurons (Krashia et al., 2017). Moreover, there is a higher percentage of *I*_h_-negative dopaminergic neurons in the VTA (Masi et al., 2015). The *I*_h_ blocker ZD7288 (<100 μM) inhibited firing activity in the SNc, but this effect was negligible in the VTA of both mice and rats (Neuhoff et al., 2002; Khaliq and Bean, 2010; Krashia et al., 2017). It is worth noting that unilateral ZD7288 injection into the SNc of adult rats prominently increased the number of apomorphine-induced rotations and reduced the immunofluorescence intensity of tyrosine hydroxylase-positive neurons. These effects were not observed in VTA neurons (Carbone et al., 2017).

Notably, *I*_h_ differentially regulates the responsiveness to excitatory synaptic inputs in SNc and VTA neurons. MPP^+^ increased the temporal summation of evoked eEPSPs in SNc neurons *via* the inhibition of *I*_h_ (Masi et al., 2013). Direct pharmacological suppression of *I*_h_ through ZD7288 (50 μM) also increased the decay time and amplitude of eEPSPs, and this response was more prominent in SNc than in VTA neurons (Masi et al., 2015). ZD7288 also significantly reduced the amplitude of evoked inhibitory post-synaptic potentials (eIPSPs) (Carbone et al., 2017). Therefore, *I*_h_ blockage likely enhances synaptic excitability through a dual mechanism in SNc dopaminergic neurons, specifically, by potentiating excitatory inputs and weakening inhibitory inputs. More importantly, increased excitatory synaptic transmission led to the amplification of SCRs in SNc neurons, while this response was minimally affected in VTA neurons (Carbone et al., 2017). In addition, low intracellular ATP caused a significant negative shift in the *I*_h_ activation curve (Carbone et al., 2017), suggesting that *I*_h_ dysfunction may be linked to the mechanisms that trigger PD, such as mitochondrial failure and ATP depletion, and that *I*_h_ dysfunction may act in concert with SNc-specific synaptic connectivity to promote selective vulnerability (Masi et al., 2015).

**FIGURE 1:**
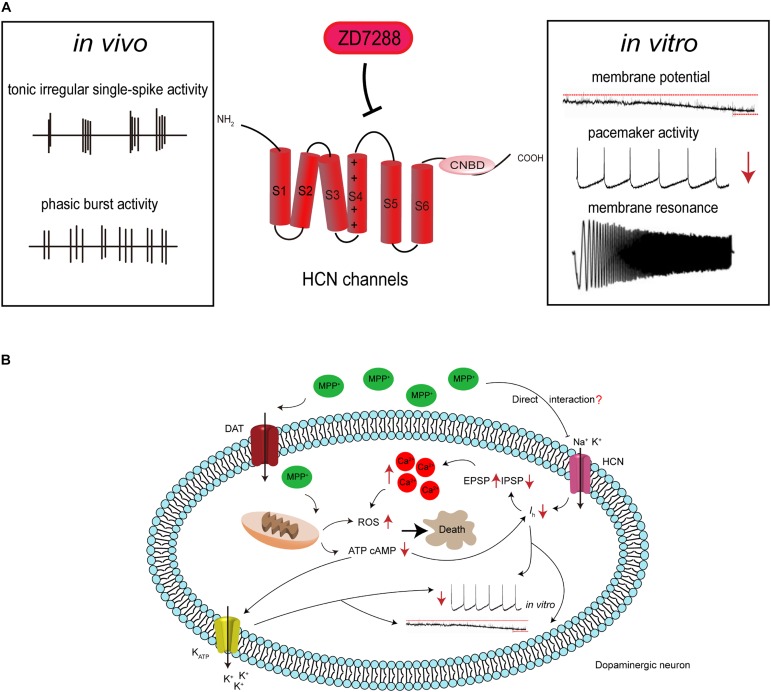
Hypothetical HCN channel-related pathogenic cascade in SNc dopaminergic neurons in PD. **(A)** HCN channels modulate the electrophysiological activities of SNc dopaminergic neurons. Dopaminergic neurons display tonic irregular single-spike firing and phasic burst firing *in vivo*, and slow, regular, pacemaker activity *in vitro*. HCN channels blockade with ZD7288 reduces the amplitude of *I*_h_, leading to cell membrane hyperpolarization, decreased firing activity, or even increased burst firing *in vitro*. HCN channels also regulate neuronal oscillatory activity. **(B)** Proposed mechanism for the involvement of HCN channels in the neurotoxic effects of MPP^+^. MPP^+^ accumulates in mitochondria, where it inhibits complex I, causing ATP depletion, increased ROS formation, and oxidative stress. The decreased cellular ATP and cAMP concentration leads to the opening of K-ATP channels and inhibition of HCN channels. This results in hyperpolarization of the cell membrane and reduction in the spontaneous firing of dopaminergic neurons. MPP^+^ is also speculated to directly interact with HCN channels, causing *I*_h_ inhibition. This further leads to the amplification of SCRs by potentiating EPSP and depressing IPSP, which results in an imbalance of intracellular calcium homeostasis. This in turn potentiates oxidative stress and ultimately leads to cell death.

Based on the evidence mentioned above, we proposed a model of the pathogenic cascade in SNc neurons during PD progression (Figure 1): with the participation of a causative agent, mitochondrial dysfunction results in a decrease in intracellular ATP levels, which in turn inhibits ATP-dependent *I*_h_. MPP^+^ may also directly interact with HCN channels, causing the inhibition of *I*_h_, and thus reducing spontaneous firing. This leads to the amplification of SCRs by potentiating EPSP and depressing IPSP, which ultimately results in an imbalance of intracellular calcium homeostasis and selective degeneration of SNc dopaminergic neurons.

Importantly, some of the above results, such as the ability of *I*_h_ to affect EPSP and EPSP-driven SCRs, were obtained using the channel blocker ZD7288. It has previously been suggested that ZD7288 induced a *I*_h_-independent reduction of synaptic transmission in hippocampal neurons (Vivien and Castillo, 2002), but this phenomenon has not been reported in SNc and VTA neurons. Therefore, the mechanism of action of ZD7288 in SNc synaptic transmission requires further verification to support our hypothesis.

## Putative Contribution of HCN Channels to Inflammatory Processes in PD

A common hallmark of neurodegenerative diseases is the presence of neuroinflammation (Griffin et al., 1998; Pott Godoy et al., 2008; Deguise and Kothary, 2017; Liu and Wang, 2017). Reactive microglia, which is the main glial cell type that participates in the inflammatory response in the brain, was first revealed in the SNc of postmortem human brain tissue as early as in 1988 (McGeer et al., 1988). Elevated cytokine IL-1β and TNF-α were detected in the SNc and striatum of PD patients and animal models (Nagatsu et al., 2000; Bartels and Leenders, 2007). Activated microglial cells secrete pro-inflammatory cytokines and produce ROS, and these inflammatory factors in turn induce the activation of astrocytes, which could not promote neuronal synapse formation, but rather released toxic factors that caused neuronal damage (De Virgilio et al., 2016; Fahmy et al., 2019).

Neuroinflammation has been reported to target specific sets of channel proteins, including HCN channels. To date, there is no report that relates the possible role of HCN channels to PD-related neuroinflammation. However, in non-PD-related brain regions, *in vivo* elevation of type I IFNs by viral brain infection or acutely applied recombinant type I IFNs to rat slices has been shown to reduce HCN1-mediated *I*_h_ in cortical pyramidal neurons (Stadler et al., 2014). More importantly, IFN-β hyperpolarized the cell membrane, reduced neuronal resonance, and modulated spontaneous EEG slow-wave activity depending on the presence of HCN1 and reversibly altered the physiological responses of cortical neuronal networks (Stadler et al., 2014). Similarly, a recent study by Federica and colleagues showed that bilateral stereotactic injection of LPS into rats’ lateral ventricles could reduce HCN1 and TRIP8b protein levels, as well as *I*_h_ amplitude and kinetics in CA1 pyramidal neurons (Frigerio et al., 2018). Moreover, LPS treatment hyperpolarized the resting membrane potential, increased the input resistance, reduced resonance properties, and increased temporal summation of synaptic inputs, which was consistent with the inhibition of *I*_h_. These data indicated that neuroinflammation can modulate the electrophysiological properties of neurons by affecting HCN channels and the related *I*_h_ current. Given the critical role of HCN channels in neuroinflammation, we boldly speculate that HCN channels could be involved in the progression of PD and even other neurodegenerative diseases *via* neuroinflammatory regulation.

## Putative Contribution of HCN Channels to the Non-Motor Symptoms in PD

Patients with PD are usually accompanied by numerous non-motor symptoms, such as depression, olfactory loss, sleep abnormalities, and constipation. To date, no studies are available concerning the possible impact of HCN channels on the non-motor symptoms of PD. However, growing evidence suggests that HCN channels and their auxiliary subunit TRIP8b play an important role in the action of antidepressant and depression. Mice with reduced *I*_h_ in the hippocampal CA1 region resulting from whole-brain deletion of TRIP8b, HCN1, or HCN2 all demonstrated significant resistance to multiple tests of behavioral despair with high predictive validity for antidepressant efficacy (Lewis et al., 2011). In TRIP8b knockout mice, restoring TRIP8b expression in the dorsal CA1 region enhanced HCN protein expression and reversed the antidepressant-like behaviors (Han et al., 2017; Lyman et al., 2017). Kim et al. (2012) further observed that rats infused with lentiviral shRNA-HCN1 in the dorsal hippocampal CA1 region displayed antidepressant- and anxiolytic-like behaviors associated with widespread enhancement of hippocampal activity. Similar result was also confirmed in CUS, a widely accepted model for major depressive disorder. Deletion of HCN1 by shRNA-HCN1 in the dorsal CA1 region prevented the CUS-induced behavioral deficits (Kim et al., 2018).

Multiple lines of evidence implicate dysregulation in the brain’s reward neural circuit in depression. Dysfunction of the dopamine reward system is thought to contribute to a hedonia and the loss of motivation common in depression (Berton et al., 2006; Nestler and Carlezon, 2006; Tye et al., 2013). *I*_h_ has also been reported to be increased in dopaminergic neurons of VTA in mice under CSDS, a well-established rodent model of depression (Cao et al., 2010; Friedman et al., 2014). However, *I*_h_ has been noted to be decreased in dopaminergic neurons of VTA in CMS, which is also a widely used depression model. A knockdown of HCN2 by lentiviral shRNA-HCN2 shRNA in the VTA produced depressive- and anxiety-like behavior, and overexpression of HCN2 in the VTA prevented the development of CMS-induced depressive-like behavior (Zhong et al., 2018). Thus, different HCN isoforms in different brain regions may play distinct roles in regulating depressive behavior.

In addition to the possible role of HCN channels and their auxiliary subunit in depression-like behavior, a possible link between HCN channels and the olfactory, sleep, and gastrointestinal functions in animal models is also noteworthy (Sun et al., 2003; Fried et al., 2010; Alicia et al., 2012; Wang et al., 2012; Shahi et al., 2014; Hu et al., 2016; Fisher et al., 2018). Because of the important diagnostic and therapeutic value of these non-motor symptoms in PD, perhaps the above studies provide a new perspective for further exploration of PD.

## HCN Channels and AD

Alzheimer’s disease is the most common neurodegenerative disease and is characterized by neuropsychiatric symptoms such as progressive memory impairment, cognitive dysfunction, personality changes, and language disorders, which seriously affect the patient’s quality of life (Gotz et al., 2018). The accumulation of the β-amyloid peptide (Aβ) within the brain and hyperphosphorylated and cleaved forms of the microtubule-associated protein tau are two key pathological features of AD (O’Brien and Wong, 2011). Lesions caused by the accumulation of these proteins mainly affect the hippocampus, the associative cortices, and subcortical structures (Braak et al., 2011). HCN channels are widely distributed in these regions and may participate in the etiology of AD by affecting neuronal excitability and regulating Aβ generation (Cirrito et al., 2005; Saito et al., 2012).

## The Relationship Between HCN Channels and Age

Changes in HCN channels in AD-associated brain regions are closely related to age. Vasilyev and Barish (2002) published a pioneering study investigating the postnatal development of *I*_h_ in mouse hippocampal pyramidal neurons. Both *I*_h_ amplitude and HCN channels immunoreactivity appeared to increase with age from postnatal 1 to 20 days in CA1 and CA3 hippocampal pyramidal neurons. In addition, *I*_h_ activation became progressively more rapid over the 1- to 20-day interval (Vasilyev and Barish, 2002). Subcellular HCN channel distribution also varied with age. HCN1 expression was found in the dendrites of rat hippocampal pyramidal neurons 2 days after birth and remained the only subtype present in dendrites until 2 weeks after birth. HCN2 was clearly distributed in the neuronal soma of rat hippocampal pyramidal neurons neonatally and was detected in the dendrites of hippocampal pyramidal neurons from the third week (Brewster et al., 2007; Bender and Baram, 2008). As rat CA1 pyramidal neurons matured, HCN1 expression prominently increased, becoming the primary HCN channel subtype (Brewster et al., 2007). This information suggests that the different expression patterns of HCN channels and *I*_h_ between immature and mature neurons may promote changes in neuronal excitability, which affect neuronal physiology and possibly lead to a pathological state.

HCN1 levels have been described to decrease dramatically in the temporal lobe of cynomolgus monkeys during aging, and be also significantly diminished in the temporal lobe of sporadic AD patients (Saito et al., 2012). The latest evidence demonstrated that the HCN1l and its auxiliary subunit, TRIP8b, were distally enriched in CA1 pyramidal neurons in both WT and 1-month-old ADTg mice but absent in ADTg mice at 12 and 24 months. The neuronal sag and rebound slope, as subthreshold voltage signatures, changed and deteriorated with age (Musial et al., 2018), and trafficking the HCN1 channel to distant dendrites restored abnormal subthreshold voltage signaling.

## Role of HCN Channels in the Regulation of Aβ Generation

Aβ is the main component of senile plaques, and its deposition may be a common pathway for all causative factors leading to AD. Aβ is generated by sequential cleavage of the transmembrane amyloid-β precursor protein (APP) by β-secretase and the γ-secretase complex. γ-secretase may cleave at either of two sites, forming two different lengths of Aβ including Aβ40 and the more neurotoxic Aβ42 (Selkoe, 2002; O’Brien and Wong, 2011). An increase in neuronal activity could enhance the production of Aβ (Kamenetz et al., 2003; Cirrito et al., 2005, 2008). Given the critical role of HCN channels in neuronal excitability, correlations between HCN channels and Aβ generation have long been suspected. Recent findings showed enhanced Aβ generation in entorhinal cortex after blockade of HCN1, as well as in global HCN1^-/-^ mice. In contrast, overexpression of HCN1 in Neuro2a cells decreased Aβ generation, whereas inhibition of the overexpressed HCN1 channel activity restored the level of Aβ production (Saito et al., 2012). HCN1^-/-^ mice had a significantly higher resting membrane potential and input resistance measured from responses to either negative or positive current steps, indicating that loss of the HCN1 subunit enhanced neuronal excitability in entorhinal cortex (Nolan et al., 2007). Thus, HCN1 may be involved in Aβ generation by regulating neuronal excitability. Eslamizade et al. (2015) further observed that rats with injection of Aβ peptides into the frontal cortex exhibited decreased excitability in hippocampal pyramidal neurons, which was caused by upregulation of *I*_h_ mediated *via* increased HCN1 mRNA.

In addition to relying on alterations in neuronal excitability, HCN1-mediated Aβ generation was also influenced by X11. The latter is an adaptor protein which binds to HCN channels and regulates the trafficking and metabolism of APP (Borg et al., 1998; Ho et al., 2002; Kimura et al., 2004; Rogelj et al., 2006). Saito et al. found that *I*_h_ amplitude and density decreased sharply in the entorhinal cortex of mice lacking both X11- and X11L. Together with X11 and X11L, APP and HCN1 were co-immunoprecipitated from the entorhinal cortex. Therefore, HCN1 may form a complex with APP and X11 or X11L to regulate the generation of Aβ (Saito et al., 2012).

**FIGURE 2:**
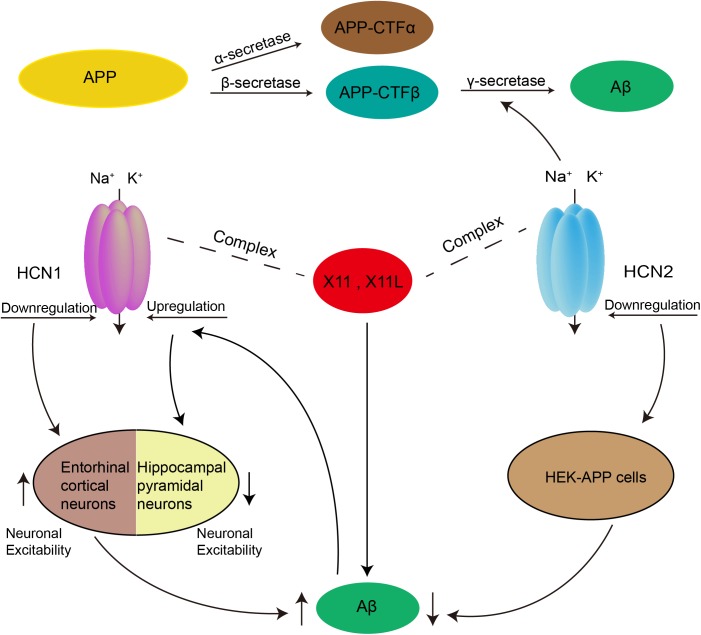
Possible role of HCN channels in the generation of Aβ. Aβ is produced by two proteolytic enzymes, β- and γ-secretase. Downregulation of HCN1 channel could enhance the production of Aβ by increasing neuronal excitability in entorhinal cortex. Rats with injection of Aβ peptides into the frontal cortex exhibited decreased excitability in hippocampal pyramidal neurons, which was caused by upregulation of *I*_h_ mediated *via* increased HCN1. In addition, both HCN1 and HCN2 channels were able to form a complex with X11 or X11L proteins to regulate Aβ generation. The HCN2 channel can also participate in the regulation of Aβ production as a γ-secretase-associated protein, and by affecting APP maturation *via* modulation of the glycosylation of APP.

As a γ-secretase-associated protein, the HCN2 channel could also participate in the regulation of Aβ production. Silencing HCN2 in HEK cells overexpressing wild-type APP (HEK-APP cells) led to a prominent decrease in Aβ40 and Aβ42, as well as a reduction in α-secretase and β-secretase cleavage. In addition, while the FL-APP levels did not change significantly, its glycosylation decreased in HEK-APP cells after silencing of HCN2 (Frykman et al., 2016). Thus, HCN2 could affect APP maturation by modulating the glycosylation of APP. Previous work has shown that glycosylated APP was reduced in Neuro2a cells overexpressing X11L (Saito et al., 2011). Interestingly, the HCN2 channel formed a complex with X11L in rat brain and in a heterologous expression cell system (Kimura et al., 2004). Thus, HCN2 may also regulate the generation of Aβ by forming a complex with X11L (Figure 2).

## Role of HCN Channels in Learning and Memory of Relevance to AD

Progressive learning and memory impairment is a major clinical symptom of AD. These changes have been primarily linked to dysregulated Ca^2+^ signaling (Bezprozvanny and Mattson, 2008; Berridge, 2011, 2014). Previous work has shown that high HCN1 expression may interfere with the generation of Ca^2+^ spikes in hippocampal CA1 pyramidal neurons (Tsay et al., 2007). In hippocampal pyramidal neurons from HCN1^-/-^ mice, the amplitude and duration of distal dendritic Ca^2+^ events have been found to be enhanced. This has also been observed after a blockade of HCN channels with ZD7288 (10 μM). This effect was attributed to a reduction in *I*_h_, which resulted in resting membrane hyperactivation and increased input resistance (Tsay et al., 2007). This resembles the previously mentioned amplification of SCRs induced by blockade of *I*_h_ in the SNc (Carbone et al., 2017).

The role of HCN channels in learning and memory has also been examined directly in gene knockout mice (Nolan et al., 2003, 2004; Matt et al., 2011; Stieglitz et al., 2017). Nolan et al. first explored the role of HCN1 in motor learning and memory. Global HCN1^-/-^ mice showed remarkable deficits in motor learning and memory, whereas those with HCN1^f/f,cre^ did not have this deficit. These defects in motor learning and memory were hypothesized to result from the loss of HCN1 in cerebellar Purkinje cells, a key component of the cerebellar circuit (Nolan et al., 2003). Although the HCN1 did not participate in the spontaneous discharge activity of Purkinje cells, HCN1 stabilizes the integration characteristics of Purkinje cells by mediating the depolarizing inward current that counteracts hyperpolarizing inputs which would otherwise push the membrane potential below the threshold for spontaneous spiking. This mechanism would allow Purkinje cells to maintain a constant input–output relationship independently of the effects of previous inputs to the cerebellum (Nolan et al., 2003).

Later, Nolan et al. (2004) investigated the role of HCN1 in spatial learning and memory. In contrast to the results mentioned above, they found that HCN1^-/-^ mice had enhanced spatial learning and memory (Nolan et al., 2004). Theta activity, which plays an important role in coding and storing spatial information, was selectively enhanced in hippocampal CA1 pyramidal neurons both in HCN1^-/-^ and HCN1^f/f,cre^ mice. Moreover, deletion of HCN1 from forebrain neurons enhanced hippocampal-dependent learning and memory, as well as LTP at the direct perforant path input to the distal dendrites of CA1 pyramidal neurons (Nolan et al., 2004). These results demonstrate that the same ion channel may have distinct functional roles in different forms of learning and memory, depending on the cell environment and the neuronal circuitry in which the channel is involved.

Matt et al. (2011) further found that LTP was significantly increased in the perforant path of HCN2^-/-^ mice. In contrast to HCN1^f/f,cre^ mice, LTP was not enhanced in mice with HCN2 selectively knocked out in hippocampal pyramidal neurons (HCN2^PyrKO^). Assessment of the amplitude and frequency of spontaneous inhibitory postsynaptic potentials (sIPSP) showed that the inhibitory interneurons were damaged in HCN2^-/-^ mice. LTP was remarkably enhanced when HCN2 was deleted from interneurons (Matt et al., 2011). Therefore, HCN2 appears to act on inhibitory interneurons in the hippocampal CA1 area to regulate LTP.

Interestingly, recent experimental results showed that global HCN3^-/-^ mice had no significant motor or spatial learning deficits in comparison with control mice (Stieglitz et al., 2017), suggesting that different subtypes of HCN channels may play different roles in learning and memory.

Notably, in an AD-related CCH animal model, HCN channels were also reported to be involved in spatial learning and memory (Li et al., 2014; Luo et al., 2015). CCH can promote learning and memory impairment and is a common risk factor for AD (de la Torre, 2004, 2010; Kalaria et al., 2012). Rats with 2VO also developed stable and long-term impairment of spatial learning and memory. Luo et al. (2015) found that rats, in the progressive phase of CCH, showed differentially altered HCN1 and HCN2 in the rat hippocampal CA1 region, and the HCN2/HCN1ratio increased throughout the process of chronic hypoperfusion. Specifically, the expression of HCN1 surface subunits was decreased at 4 weeks of 2VO, but exhibited no significant change at 8 or 12 weeks. In contrast to HCN1, HCN2 surface expression was increased at all time points of ischemia (Luo et al., 2015). In normal conditions, HCN1 subunit expression is more abundant than HCN2 in the hippocampal CA1 region. Therefore, the HCN1 subunit is more likely to form homologous channels. However, during the CCH process, due to the continuous increase of HCN2 subunits, the probability that HCN1 subunits will form heterologous channels with HCN2 subunits increases. Since the properties of homologous and heterologous channels differ, the hippocampal synaptic plasticity attributed to HCN channels is altered, which in turn leads to changes in spatial learning and memory. Thus, they proposed that in the early stages of CCH, the impairment of spatial motor memory in rats may be caused by common changes of HCN1 and HCN2, and later, the disorder was attributed to the upregulation of HCN2 expression. A similar phenomenon was also observed by Li et al. (2014) in the hippocampal CA1 area in rats after 5 weeks of 2VO, with reduced HCN1 expression and increased HCN2 expression. Moreover, Li et al. (2014) found that restoring the surface expression of HCN1 and HCN2 could ameliorate the impairment of spatial learning and memory. Together, these studies on learning and memory suggest novel avenues of research into the pathogenesis of AD (Table 2).

**Table 2:** Roles of HCN channels in learning and memory of relevance to AD.

Models	Main findings	References
HCN1^-/-^ mice	As *I*_h_ decreases, the resting membrane potential became hyperactivated and input resistance increased significantly, resulting in further enhancement of the amplitude and duration of distal dendritic Ca^2+^ events.	Tsay et al., 2007
HCN1^-/-^ mice/HCN1*^f/f,cre^* mice	Theta activity was selectively enhanced in hippocampal CA1 pyramidal neurons.	Nolan et al., 2004
HCN1*^f/f,cre^* mice	LTP was significantly enhanced at the direct perforant path input to the distal dendrites of CA1 pyramidal neurons, as was spatial learning and memory.	Nolan et al., 2004
HCN2^-/-^ mice	LTP was significantly enhanced.	Matt et al., 2011
Mice with deletion of HCN2 from interneurons	LTP was significantly enhanced.	Matt et al., 2011
HCN3^-/-^ mice	There were no significant deficits in motor learning and spatial learning in comparison with control mice.	Stieglitz et al., 2017
2VO rats	Rats displayed a prolonged time to swim to the platform and altered expression patterns of HCN1 and HCN2 in the hippocampal CA1 area. Spatial learning and memory impairment could be improved when restoring the expression of HCN1 and HCN2.	Li et al., 2014; Luo et al., 2015


*HCN1^-/-^, HCN1 knockout; HCN1^*f/f,cre*^, forebrain-restricted knockout; HCN2^-/-^, HCN2 knockout; HCN3^-/-^, HCN3 knockout; LTP, long-term potentiation; 2VO, permanent bilateral occlusion of the common carotid arteries.*

## Role of HCN Channels in Other Neurodegenerative Diseases

### HCN Channels and ALS

Amyotrophic lateral sclerosis is a universally fatal neurodegenerative disease characterized by progressive loss of corticospinal neurons, brainstem motor neurons, and spinal motor neurons, which leads to progressive weakness and paralysis. A recent study found that vesicle associated membrane protein B (VAPB), which modulates the surface expression and cellular localization of HCN1 and HCN2 channels, exhibited decreased expression in motor neurons of ALS8 patients, a typical form of familial ALS, as well as in the motor neurons of sporadic ALS patients (Anagnostou et al., 2010; Mitneneto et al., 2011; Silbernagel et al., 2018). Silbernagel et al. (2018) reported that HCN2 and VAPB were co-localized in both HCN2-transfected HeLa cells and VAPB-transfected HeLa cells. However, this co-localization was not observed in HeLa cells transfected with the VAPB^P56S^ mutant (Silbernagel et al., 2018). Indeed, HCN2 current amplitude was noticeably decreased by VAPB modulation. Mutation or loss of VAPB led to a decrease in HCN activity and neuronal excitability in motor neurons, which are a main cell type involved in the pathogenesis of ALS (Silbernagel et al., 2018).

### HCN Channels and SMA

Spinal muscular atrophy is an autosomal recessive motor neuron disorder, characterized by progressive muscle weakness, especially in the torso and proximal limbs (Russman, 2007). Currently, only two studies have examined the correlation between HCN channels and SMA. Similarly, to ALS, a mutation in VAPB has also been identified in SMA patients (Nishimura et al., 2004), establishing a potential connection between HCN channels and SMA, as VAPB modulates HCN channels. Recently, a marked enhancement of *I*_h_ amplitude as well as HCN channel expression was observed in the spinal cord and sciatic nerves of SMA mice. Furthermore, treatment with ZD7288 appeared to reduce early mortality, improve motor function, and restore neuromuscular junction architecture in SMA mice (Lai et al., 2018). These results provide initial evidence that HCN channels may play a role in SMA pathophysiology and could be a novel target for SMA treatment.

## Conclusion

HCN channels are key regulators of neuronal excitability and network activity within the nervous system. Increasing evidence supports a model in which modifications in their physiological function contributes to the pathogenic mechanisms of several neurodegenerative diseases, implying that they may be a potential therapeutic target. This review, albeit simplistic, summarizes much of the research aimed at understanding the roles of HCN channels in animal models of PD, AD, and other neurodegenerative diseases, as well as in patients. To date, studies have reported reduced HCN1 expression in AD patients (Saito et al., 2012) as well as mutation or loss of VAPB, an HCN regulator protein in ALS (Anagnostou et al., 2010; Mitneneto et al., 2011) and SMA patients (Nishimura et al., 2004). However, gene variants/mutations in HCN channels or their regulatory proteins have not been identified in human patients with other neurodegenerative disorders. Presently, research concerning the role of HCN channels in neurodegenerative diseases is in its infancy, with a lack of probing for them as candidate genes. Interestingly, recent work has identified a mutation in the HCN2 gene, and augmentation of *I*_h_ in patients with genetic epilepsy with febrile seizures plus. This study provides evidence for the possible involvement of HCN channel mutations in familial forms of human epilepsy (Dibbens et al., 2010). Therefore, further research on the variations of HCN and their regulatory proteins may be an exciting area for future research, especially in patients with neurodegenerative diseases.

## Author Contributions

XC wrote the manuscript. JW, HJ, LS, and JX approved and revised the final manuscript.

## Conflict of Interest Statement

The authors declare that the research was conducted in the absence of any commercial or financial relationships that could be construed as a potential conflict of interest.

## Abbreviations

2VO: permanent bilateral occlusion of the common carotid arteries
6-OHDA: 6-hydroxydopamine
Aβ: β-amyloid peptide
AD: Alzheimer’s disease
ALS: amyotrophic lateral sclerosis
APP: amyloid-β precursor protein
calbindin: calcium-binding protein
cAMP: cyclic adenosine monophosphate
CCH: chronic cerebral hypoperfusion
Cm: capacitance values
CMA: chronic mild unpredictable stress
CSDS: chronic social defeat stress
CUS: chronic unpredictable stress
DAT: dopamine transporter
EPSPs: excitatory post-synaptic potentials
FL-APP: full-length APP
GP: globus pallidus
GPe: external globus pallidus
GPi: interior globus pallidus
HCN channels: hyperpolarization-activated cyclic nucleotide-gated channels
HCN1^-/-^: HCN1 knockout
HCN1^f/f,cre^: forebrain-restricted knockout
HCN2^-/-^: HCN2 knockout
HCN3^-/-^: HCN3 knockout
IFNs: interferons
IL-1β: interleukin 1β
IPSPs: inhibitory post-synaptic potentials
K-ATP: ATP-sensitive potassium channels
LPS: lipopolysaccharide
LTP: long-term potentiation
MPP^+^: 1-methyl-4-phenylpyridinium
MPTP: 1-methyl-4-phenyl-1,2,3,6-tetrahydropyridine
PD: Parkinson’s disease
Rm: membrane resistance
ROS: reactive oxygen species
SCRs: somatic calcium responses
sIPSC: spontaneous inhibitory postsynaptic potentials
SMA: spinal muscular atrophy
SNc: substantia nigra pars compacta
STN: subthalamic nucleus
TNF-α: tumor necrosis factor-α
TRIP8b: Rab8b-interacting protein
VAPB: vesicle-associated membrane protein B
VTA: ventral tegmental area
X11L: X11-like protein
